# The Interplay between the Hippocampus and Amygdala in Regulating Aberrant Hippocampal Neurogenesis during Protracted Abstinence from Alcohol Dependence

**DOI:** 10.3389/fpsyt.2013.00061

**Published:** 2013-06-27

**Authors:** Chitra D. Mandyam

**Affiliations:** ^1^Committee on the Neurobiology of Addictive Disorders, The Scripps Research Institute, La Jolla, CA, USA

**Keywords:** chronic ethanol, vapor induced dependence, self-administration, subgranular zone, hippocampus, BrdU

## Abstract

The development of alcohol dependence involves elevated anxiety, low mood, and increased sensitivity to stress, collectively labeled negative affect. Particularly interesting is the recent accumulating evidence that sensitized extrahypothalamic stress systems [e.g., hyperglutamatergic activity, blunted hypothalamic-pituitary-adrenal (HPA) hormonal levels, altered corticotropin-releasing factor signaling, and altered glucocorticoid receptor signaling in the extended amygdala] are evident in withdrawn dependent rats, supporting the hypothesis that pathological neuroadaptations in the extended amygdala contribute to the negative affective state. Notably, hippocampal neurotoxicity observed as aberrant dentate gyrus (DG) neurogenesis (neurogenesis is a process where neural stem cells in the adult hippocampal subgranular zone generate DG granule cell neurons) and DG neurodegeneration are observed in withdrawn dependent rats. These correlations between withdrawal and aberrant neurogenesis in dependent rats suggest that alterations in the DG could be hypothesized to be due to compromised HPA axis activity and associated hyperglutamatergic activity originating from the basolateral amygdala in withdrawn dependent rats. This review discusses a possible link between the neuroadaptations in the extended amygdala stress systems and the resulting pathological plasticity that could facilitate recruitment of new emotional memory circuits in the hippocampus as a function of aberrant DG neurogenesis.

## Neurogenesis in the Adult Dentate Gyrus

**Figure 1:**
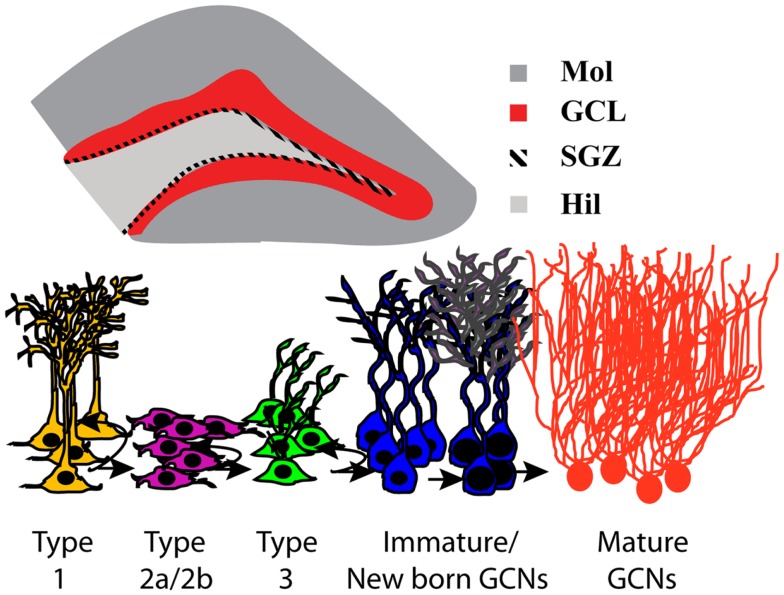
**Neurogenesis in the subgranular zone of the hippocampus**. Schematic representation of the coronal view of the hippocampus region; magnification of the DG region in a coronal view −3.6 mm from bregma indicating the subregions of the DG and highlighting the neurogenic region; GCL in red and SGZ as the hatched area. DG, dentate gyrus; GCL, granule cell layer; Mol, molecular layer; SGZ, subgranular zone; Hil, hilus. Stages of adult hippocampal neurogenesis are indicated below the schematic of the coronal view of the hippocampus. In the DG, type 1 putative stem-like cells are slowly dividing and rarely label with the commonly used exogenous mitotic marker 5-bromo-2′-deoxyuridine (BrdU) but can be identified via morphology and staining for nestin/GFAP/Sox2. BrdU will label rapidly dividing type 2 and some type 3 cells. Type 3 cells mature and differentiate into immature granule cell neurons and migrate a short distance into the granule cell layer to become granule cell neurons and integrate into the hippocampal circuitry.

Accumulating evidence over the past four decades shows that forebrain neural stem cells populate two main areas, the subventricular zone of the lateral ventricles and subgranular zone (SGZ) of the hippocampal dentate gyrus (DG; Figure 1), where they give rise to neurons throughout adulthood. Adult neurogenesis is found in these forebrain regions in all mammalian species examined, including humans (Eriksson et al., 1998; Curtis et al., 2007), and may serve to replace cells damaged by brain disorders, such as addiction to drugs of abuse and alcohol. Whether they replace dying or diseased cells and if so to what extent are questions currently receiving intense research focus.

Adult neurogenesis in the hippocampal DG plays an important role in maintaining hippocampal plasticity. The process of neurogenesis involves stem-like precursor cells (type 1 cells) that proliferate into preneuronal progenitors (type 2 and type 3), which in turn differentiate into immature neurons and eventually mature into granule cell neurons (GCNs; Kempermann et al., 2004; Abrous et al., 2005; Figure 1). A large proportion (>80%) of hippocampal progenitors migrate a short distance to become GCNs in the DG (Kaplan and Hinds, 1977; Hastings et al., 2001), and there is evidence demonstrating functional incorporation of the newly born neurons in the DG (Gould et al., 1999; Shors et al., 2002; Aimone et al., 2006). For example, DG neurogenesis has been implicated in the maintenance of hippocampal networking (Aimone et al., 2006; Clark et al., 2012; Lacefield et al., 2012) and assists with certain behaviors that depend on the hippocampus (Feng et al., 2001; Deisseroth et al., 2004; Schmidt-Hieber et al., 2004; Kim et al., 2012) and is critical for encoding new information by facilitating the formation of new memories that assist with hippocampus-dependent behaviors (McHugh et al., 2007; Bakker et al., 2008; Clelland et al., 2009; Aimone et al., 2011; Sahay et al., 2011).

**Figure 2:**
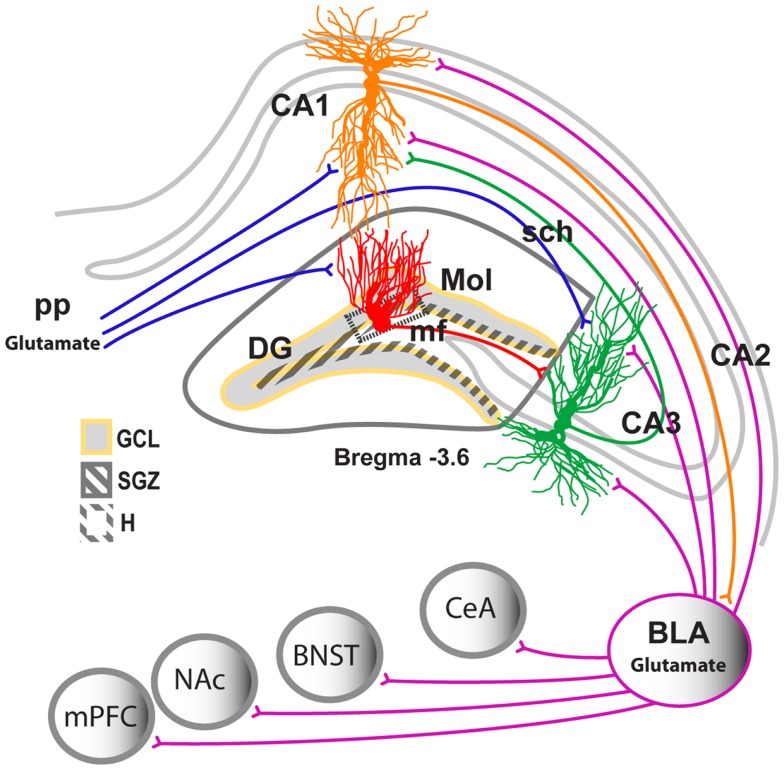
**Neuronal projections in the hippocampus**. Schematic representation of the coronal view of the hippocampus region indicating the subregions of the hippocampus and their location within the hippocampus. CA, cornu ammonis; Trisynaptic circuitry in the hippocampus is indicated with axons from the entorhinal cortex projecting unidirectionally to the apical dendrites of the hippocampal DG, CA1, and CA3 neurons (perforant path projection). DG neurons project to the apical dendrites of the CA3 pyramidal neurons (mossy fiber projection). CA3 neurons project to the apical dendrites of the CA1 neurons (Schaffer collateral projection). The CA1 neurons have bidirectional projections to and from the BLA. The BLA also sends projections to the medial prefrontal cortex (mPFC), nucleus accumbens (NAc), bed nucleus of the stria terminalis (BNST), and central nucleus of the amygdala.

Dentate gyrus neurogenesis is also strongly regulated by stress and glucocorticoids (Cameron and Gould, 1994; Mirescu and Gould, 2006; Oomen et al., 2007; Snyder et al., 2011). Conversely, DG neurogenesis regulates the secretion of glucocorticoids in response to stress (Snyder et al., 2011). This is important because the hippocampus provides negative control of the hypothalamic-pituitary-adrenal (HPA) axis, and DG neurogenesis regulates hippocampal regulation of the HPA axis (Snyder et al., 2011), although the circuitry mediating this effect is not well understood. Furthermore, the role of the glutamatergic system in the development and maintenance of DG neurogenesis is well documented (Cameron et al., 1995). For example, *N*-methyl-d-aspartate (NMDA) receptor activation reduces the proliferation of neural precursors in a normal state, and blockade of NMDA receptors increases the birth and survival of neural precursors in the DG, suggesting that neuronal inputs into the hippocampus regulate DG neurogenesis (Figure 2). Furthermore, recent evidence demonstrates compromised HPA axis activity (Richardson et al., 2008), altered glucocorticoid signaling (Vendruscolo et al., 2012), increased sensitivity to NMDA-mediated function (Becker et al., 1998; Gonzalez et al., 2001), and significant reductions in the rate of DG neurogenesis (Nixon and Crews, 2002; Richardson et al., 2009; Hansson et al., 2010) in a preclinical models of alcohol addiction and dependence. These data suggest that the normalization of alcohol-impaired DG neurogenesis during withdrawal may help reverse altered hippocampal neuroplasticity during protracted abstinence and thus may help reduce the vulnerability to relapse and aid recovery.

## Animal Models of Chronic Alcohol Exposure and Alcohol Dependence

There are several *in vitro* and *in vivo* preclinical model systems that represents various stages of alcohol intoxication, addiction, and dependence. Three models are highlighted in this review; *in vitro* organotypic hippocampal cell culture model, intragastric intubation model, and chronic ethanol vapor induced dependence (CEID) model. The incorporation of these models has allowed us to determine the toxic and neuromodulatory effects of ethanol in specific brain regions and reward systems. The *in vitro* organotypic hippocampal cell culture model is commonly used to study hippocampal excitotoxicity associated with alcoholism. The *in vitro* model harbors critical hippocampal heterogeneity that is necessary for neuron–neuron and neuron-glia interactions to occur, thus maintaining the structural and functional integrity of hippocampal circuitry and pharmacology (Gutierrez and Heinemann, 1999; Martens and Wree, 2001). Notably, the *in vitro* model has been extensively used to study the effects of chronic ethanol and withdrawal from ethanol on hippocampal neurotoxicity and excitotoxicity (Gibson et al., 2003; Prendergast et al., 2004; Wilkins et al., 2006). Studies indicate that ethanol excitotoxicity is dependent on the concentration of ethanol and duration of withdrawal after ethanol exposure. The intragastric intubation model has been widely used to study hippocampal neurotoxicity associated with alcoholism. This model produces observable signs of prodromal detoxification and physiological dependence (Majchrowicz, 1975), and these extreme signs of ethanol intoxication and dependence have been correlated with reduced neuroplasticity and enhanced neurodegeneration (Nixon and Crews, 2002; Crews and Nixon, 2009).

The CEID model of alcohol dependence links chronic ethanol exposure regimens with self-administration procedures. This model is based on the idea that dependence and the experience of withdrawal during dependence drive excessive drinking during withdrawal through altered motivational processes (e.g., negative reinforcement; O’Dell et al., 2004; Lopez and Becker, 2005; Gehlert et al., 2007; Griffin et al., 2009). The CEID model has several advantages compared with the intragastric intubation model of alcohol dependence because it causes increases in ethanol self-administration and enhanced responsiveness to environmental stimuli that lead to excessive drinking in humans (Valdez et al., 2002; O’Dell et al., 2004). Importantly, CEID produces relatively high blood alcohol levels (BALs) during a short period of time, making this approach advantageous for studying the somatic aspects, motivational aspects, and neurobiological consequences of alcohol dependence (Macey et al., 1996; Liu and Weiss, 2002, 2003; Moore et al., 2004; Budygin et al., 2007; Miki et al., 2008; Gilpin et al., 2009; Richardson et al., 2009; Zahr et al., 2009). Altogether, investigating the neurobiological effects of chronic ethanol in CEID models has helped identify other vulnerability factors that contribute to the pathology of alcoholism in humans (Macey et al., 1996; Liu and Weiss, 2002, 2003; Moore et al., 2004; Budygin et al., 2007; Miki et al., 2008; Gilpin et al., 2009; Richardson et al., 2009; Zahr et al., 2009; Hansson et al., 2010).

## Alcohol and the Morphology and Plasticity of the Hippocampus

The hippocampus is involved in ethanol reward and relapse to ethanol seeking (Koob and Volkow, 2010; Zarrindast et al., 2010), suggesting that the hippocampus contributes to several aspects of alcohol dependence and can be implicated in the phenomena linked to alcohol use disorders. For example, alcohol dependence is linked to decreased hippocampus volume (Sullivan et al., 1995; Beresford et al., 2006), altered hippocampal morphology (Bengochea and Gonzalo, 1990; Durazzo et al., 2011), and deficits in hippocampus-dependent learning and memory (Brandt et al., 1983; Glenn and Parsons, 1991; Sullivan et al., 2000a,b, 2002). Alcohol exposure also alters the functional plasticity of hippocampal neurons. For instance, acute ethanol in hippocampal slices decreases hippocampal synaptic activity [i.e., decreases NMDA and α-amino-3-hydroxy-5-methyl-4-isoxazole-propionic acid (AMPA) receptor-mediated currents and increases γ-aminobutyric acid-A (GABA_A_) receptor-mediated currents] and decreases hippocampal (CA1 and DG) long-term potentiation (LTP; Lovinger et al., 1989; Blitzer et al., 1990; Wayner et al., 1997; Weiner et al., 1999; Wright et al., 2003; Izumi et al., 2005; Fujii et al., 2008). Notably, chronic ethanol exposure also impairs hippocampal CA1 LTP through a presynaptic LTP mechanism (Durand and Carlen, 1984; Roberto et al., 2002) and produces tolerance to acute ethanol-mediated decreases in hippocampal LTP (Fujii et al., 2008), suggesting reorganization of hippocampal networking after chronic ethanol exposure. Furthermore, chronic ethanol exposure oppositely affects hippocampal synaptic activity compared with acute ethanol (increases in NMDA and decreases in GABA_A_ receptor-mediated activity) and produces tolerance to acute ethanol-mediated impairment of NMDA activity and hippocampal-dependent behaviors (Sanna et al., 1993; Wu et al., 1993; Nelson et al., 2005; Sheela Rani and Ticku, 2006; Fujii et al., 2008). These findings indicate that the cellular mechanisms that maintain hippocampal plasticity are compensated in chronic ethanol-exposed animals. These maladaptive changes could contribute to the impairment of hippocampus-dependent behaviors in alcohol-dependent animals (Lukoyanov et al., 1999; Cippitelli et al., 2010; George et al., 2012). Chronic ethanol exposure produces dendritic retraction of CA1 pyramidal neurons (McMullen et al., 1984), suggesting concomitant structural reorganization of hippocampal neurons compared with functional changes in hippocampal circuitry. Recent evidence demonstrated that ethanol exposure altered a new form of hippocampal plasticity, such as DG neurogenesis (reviewed in (Nixon, 2006; Mandyam and Koob, 2012). Ethanol exposure (i.e., intragastric intubation, two-bottle choice, ethanol liquid diet, and CEID) altered every stage of DG neurogenesis, including the proliferation, differentiation, maturation, and survival of neural stem cells (Figure 1). These effects varied by the dose, duration, and pattern of ethanol exposure and timing of ethanol exposure before labeling the neural progenitors (Nixon and Crews, 2002; Crews et al., 2004; Rice et al., 2004; He et al., 2005; Ieraci and Herrera, 2007; Richardson et al., 2009; Taffe et al., 2010; Contet et al., 2013). Therefore, the inhibitory effect of ethanol on the regenerative capacity of the adult hippocampus is now being considered a precursor for ethanol-induced neurodegeneration in the hippocampus (Nixon, 2006).

## Alcohol Exposure Produces Neurotoxicity and Excitotoxicity in the Hippocampus

Using the *in vitro* organotypic hippocampal cell culture model, it has been demonstrated that hippocampal CA1 excitotoxicity is evident after withdrawal from chronic ethanol exposure and not during ethanol exposure (Mulholland et al., 2003; Prendergast et al., 2004; Wilkins et al., 2006). Withdrawal-associated effects have been shown to be due to the release of excessive glutamate and polyamines and corresponding activation of NMDA-type receptors in the hippocampal region (Gibson et al., 2003). Importantly, ethanol studies that used the *in vitro* model indicate the importance of the glutamatergic system as a final common pathway mediating neurotoxicity and excitotoxicity. There are also *in vivo* studies that support the involvement of the glutamatergic system in ethanol-induced hippocampal neurotoxicity in chronic ethanol-exposed animals (Claus et al., 1982; Keller et al., 1983; Wilce et al., 1993; Snell et al., 1996; Wirkner et al., 1999). For example, glutamate release is increased in the hippocampus during ethanol withdrawal (Claus et al., 1982; Keller et al., 1983), and changes in glutamate levels are associated with enhanced polyamine levels in combination with an increased number of functional NMDA receptors (Davidson et al., 1993, 1995). These results suggest that increased glutamate levels may induce ethanol withdrawal hyperexcitability and lead to increased susceptibility to hippocampal excitotoxicity (Hoffman, 2003).

## Withdrawal and Protracted Abstinence from Alcohol and DG Neurogenesis

Very few studies have explored how forced withdrawal from drug exposure alters DG neurogenesis (Nixon and Crews, 2004; Nixon et al., 2008; Noonan et al., 2008; Barr et al., 2010; Hansson et al., 2010; Taffe et al., 2010; Garcia-Fuster et al., 2011; Deschaux et al., 2012; Recinto et al., 2012). Withdrawal from ethanol exposure in the intragastric intubation and CEID paradigms enhanced cell proliferation in the hippocampus (Nixon and Crews, 2004; Hansson et al., 2010), resulting in initial microglial proliferation (Nixon et al., 2008) followed by the production of immature neurons and eventual neurogenesis (Nixon and Crews, 2004). Aberrant neurogenesis during abstinence is thought to be attributable to central nervous system hyperexcitability associated with ethanol withdrawal symptomatology, such as whole-body tremors that result from the termination of ethanol exposure. However, the cellular mechanisms regulating ethanol withdrawal-induced aberrant neurogenesis in the DG have not been identified, and future mechanistic studies are needed to address the contribution of aberrant DG neurogenesis to brain changes associated with alcohol dependence.

## Withdrawal and Protracted Abstinence from Alcohol and Epileptogenesis and Neuroadaptations in the Hippocampus

As discussed earlier, both *in vitro* and *in vivo* evidence suggests that glutamatergic neurotransmission is a critical mediator of the experience-dependent synaptic plasticity that may underlie alcohol dependence. It is hypothesized that a hyperglutamatergic state in the basolateral amygdala (BLA) resulting from termination of ethanol exposure may be regulated by a variety of neuroadaptations in the extended amygdala. These alterations may regulate the plasticity in the hippocampus to produce the withdrawal hyperexcitability associated with dependence (Hoffman and Tabakoff, 1994; Tsai et al., 1995; Nixon and Crews, 2004; McCool et al., 2010; Prior and Galduroz, 2011). For example, withdrawal from ethanol, especially the termination of CEID, produces withdrawal symptomatology, manifested as increased acoustic startle reactivity and tremor activity that peaks 12–24 h post-withdrawal (Macey et al., 1996). These somatic symptoms of ethanol withdrawal seem to have an immediate effect on hippocampal plasticity. Withdrawal from CEID produces a rebound effect on the proliferation of neural progenitors that occurs 72 h after the termination of CEID. These cells propagate into aberrant immature GCNs during protracted abstinence (Hansson et al., 2010). Notably, pilocarpine-induced status epilepticus also produces abnormal proliferation of neural progenitors in the DG that is evident 72 h after seizure activity (Parent et al., 1997). This is a timeframe comparable to ethanol withdrawal-induced alterations. In addition to the alterations in DG neural progenitors, both epileptic activity and withdrawal from CEID have other common cellular and molecular neuroadaptations in the hippocampus. Particularly interesting is the increases in NMDA receptor 2B (NR2B) subunit expression in the hippocampus during CEID (Pian et al., 2010) and CRF levels in the hippocampus during withdrawal (Criado et al., 2011). These changes parallel the increased NR2B subunit and CRF expression in the hippocampus during epileptogenesis (Smith et al., 1997; Frasca et al., 2011). Altogether, it appears that the hyperactivity stemming from the neurocircuitry underlying ethanol withdrawal-induced kindling-like behaviors causes a hyperglutamatergic state and produces hippocampal excitotoxicity, which may be decisive factors for the maintenance of long-term dependence (Baram et al., 1992; Smith et al., 1997; Wilkins et al., 2006; Frasca et al., 2011; Prior and Galduroz, 2011).

## Withdrawal and Protracted Abstinence from Alcohol Alter HPA Axis and Glucocorticoid Receptor Signaling

Animals made dependent by CEID or liquid diet procedures have attenuated (opposing) basal stress hormone levels (adrenocorticotropic hormone and corticosterone) compared with non-dependent drinking animals (enhanced stress hormone levels). It has been demonstrated that the blunted stress response is a consequence of chronic ethanol exposure (Zorrilla et al., 2001; Richardson et al., 2008). Importantly, the findings from animal studies are consistent with clinical studies that link maladaptive HPA axis function with alcoholism, including a reduced ability to cope with stress and negative correlations between cortisol and craving and relapse in alcoholics (Lovallo et al., 2000; O’Malley et al., 2002). Although the precise mechanism underlying the attenuated stress response is unknown, several studies have implicated activation of CRF systems in the extended amygdala in the dysregulation of the stress system associated with dependence (Wand, 2005; Koob, 2008). Furthermore, enhanced glucocorticoid receptor (GR) levels in the extended amygdala during protracted abstinence have been demonstrated in dependent animals. Such associated changes in the GR system could play a mechanistic role in the sensitivity to stress/reward and relapse associated with alcohol dependence (Vendruscolo et al., 2012). However, the functional significance of altered GR system in mediating blunted stress responses in alcohol dependence is unknown.

## Relationship between Ethanol-Induced Neuroadaptive Changes in the Amygdala and Aberrant DG Neurogenesis

The aberrant stimulation of cell proliferation in the DG during withdrawal from chronic ethanol exposure has been demonstrated in the *in vitro* organotypic hippocampal cell culture model (Wilkins et al., 2006), intragastric intubation model (Nixon and Crews, 2004; Nixon et al., 2008), and CEID model (Hansson et al., 2010). Further mechanistic experiments that used the intragastric intubation model demonstrated that observable withdrawal signs correlated with increases in cell proliferation. However, rescuing the observable withdrawal symptoms with diazepam did not normalize the cell proliferation effects (Nixon and Crews, 2004). This suggests that withdrawal-induced enhanced proliferation is not secondary to the physiological withdrawal experienced by the animal but may be related to the neuroadaptations linked to the negative affect symptoms associated with alcohol dependence.

Possible mechanisms underlying ethanol withdrawal-induced aberrant DG cell proliferation and neurogenesis can be postulated based on the available literature. For example, the increased synthesis of hippocampal CRF during withdrawal (Criado et al., 2011) might promote excitatory activity and lead to BLA hyperexcitability, which in turn may increase the level of CRF at critical hippocampal synapses (Figure 2). Such a mechanism would further enhance excitability in a positive-feedback manner in the hippocampus during ethanol withdrawal (Baram and Hatalski, 1998; Hollrigel et al., 1998; Chen et al., 2004). Increased CRF synthesis in the hippocampus could be due to decreased hippocampal inhibitory GABA activity seen during ethanol withdrawal (Frye et al., 1983; Fujii et al., 2008). The excitatory effect of CRF on DG neurons in the hippocampus may occur indirectly through CRF-induced activation of excitatory inputs into the hippocampus to cause DG hyperexcitability (Hollrigel et al., 1998). Epileptogenic studies suggest that excitatory glutamatergic projections from the BLA are implicated in DG excitotoxicity and hyperexcitability (Baram et al., 1992; Freund and Buzsaki, 1996; Smith et al., 1997; Hollrigel et al., 1998; Yan et al., 1998; Wang et al., 2000). Notably, most of the projection neurons from the BLA to the hippocampus are glutamatergic and express CRF_1_ receptors. Specific knockdown of CRF_1_ in BLA glutamatergic neurons produces anxiolytic-like effects (Refojo et al., 2011). Furthermore, the CRF system in the BLA is hypothesized to be recruited by chronic kindling cycles of ethanol exposure/withdrawal (Baram et al., 1992; Rimondini et al., 2003; Breese et al., 2004; Knapp et al., 2004; Overstreet et al., 2004; O’Dell et al., 2004) and mediate the motivating, negative affective symptoms of both acute and protracted abstinence from ethanol. Protracted abstinence from CEID enhances BLA CRF_1_ levels (Sommer et al., 2008), suggesting that BLA sensitivity to CRF increases in a kindling-like fashion during withdrawal (Sajdyk et al., 1999; Sajdyk and Gehlert, 2000; Rainnie et al., 2004). Recent functional studies demonstrated that DG neurogenesis is regulated by BLA neuronal activity (Kirby et al., 2012), and a kindling procedure specifically in the BLA produced aberrant DG neurogenesis, which resulted from the altered expression of cell differentiation factors in the DG neurogenic niche (Fournier et al., 2010). Therefore, increases in CRF in the extended amygdala could produce secondary effects on DG neurogenesis via the BLA. These alterations could be hypothesized to be regulated by corticosterone levels (Makino et al., 1994).

A related mechanism for ethanol withdrawal-induced increases in cell proliferation and DG neurogenesis could be ethanol withdrawal-induced blunting of corticosterone levels (Richardson et al., 2008) and corresponding increases in GR levels in the extended amygdala (Vendruscolo et al., 2012). The reduced levels of corticosterone could enhance DG proliferation and neurogenesis to assist with the hippocampal negative feedback regulation of HPA axis activity (Jankord and Herman, 2008; Snyder et al., 2011). Furthermore, it has been demonstrated that withdrawal is associated with upregulation of NMDA receptors, specifically in the hippocampus (Hoffman, 2003), which is perhaps secondary to glucocorticoid-dependent excess release of endogenous glutamate and polyamines in the hippocampus and extended amygdala (Abraham et al., 2001; Gibson et al., 2003). Although NMDA receptor activation has been shown to reduce cell proliferation in a normal state (Cameron et al., 1995), this effect is reversed during cytotoxicity (e.g., ethanol withdrawal; Wilkins et al., 2006) and could be attributable to the altered expression of NMDA receptor subunits in chronic ethanol-exposed animals compared with ethanol-naive animals (Prendergast and Mulholland, 2012; Ren et al., 2013). Altogether, specific corticosteroid-mediated neuroadaptations in the CRF system in the extended amygdala following ethanol withdrawal could produce a hyperglutamatergic state in the hippocampus, which may regulate aberrant neurogenesis in the DG. The resulting pathological plasticity could facilitate the recruitment of new GCNs into emotional memory circuits and therefore contribute to the pathology of alcohol dependence (Farioli-Vecchioli et al., 2009; Fournier et al., 2013). Future studies should seek to understand the underlying mechanism of ethanol withdrawal-induced aberrant DG neurogenesis. Such studies may help determine whether hippocampal GCNs born during withdrawal perform improper functions to inhibit regeneration in the hippocampus (excitotoxicity) and aid with recruitment of new neurons into emotional memory circuitry (negative affect).

## Conflict of Interest Statement

The authors declare that the research was conducted in the absence of any commercial or financial relationships that could be construed as a potential conflict of interest.
